# Demand for community-based Case Management in Austria - a qualitative analysis

**DOI:** 10.1186/s12912-021-00775-0

**Published:** 2022-01-04

**Authors:** Alessandra Schirin Gessl, Angela Flörl, Eva Schulc

**Affiliations:** 1 LMU Munich School of Management, Ludwig-Maximilians-Universität (LMU) München, Geschwister-Scholl-Platz 1, 80539 Munich, Germany; 2 Division of Integrated Care, Institute of Nursing Science, Department of Nursing Science & Gerontology, UMIT - Private University for Health Sciences, Medical Informatics and Technology, Hall in Tyrol, Austria

**Keywords:** Home healthcare, Community care, Focus group, nurse roles, Multi-professional practice, Case management

## Abstract

**Background:**

The number of people with complex nursing and care needs living in their own homes is increasing. The implementation of Case and Care Management has shown to have a positive effect on unmet care needs. Research on and implementation of Case and Care Management in the community setting in Austria is limited. This study aimed to understand the changes and challenges of changing care needs by mobile nurses and to evaluate the need for Case Management in mobile care organizations by investigating the evolution of mobile care nurses‘task profiles and the challenges in working in a dynamic field with changing target groups and complexifying care needs.

**Methods:**

A qualitative study with reductive-interpretative data analysis consisting of semi-structured focus groups was conducted. Community care nurses, head nurses, and managers of community mobile care units as well as discharge managers of a community hospital (*n* = 24) participated in nine qualitative, semi-structured focus groups. The recorded focus groups were transcribed and analyzed using qualitative content analysis.

**Results:**

The analysis revealed three main categories: the complexity of the case, innerinstitutional frameworks, and interinstitutional collaboration, which influence the perception of need for further development in the direction of Case and Care Management. Feelings of overwhelmedness among nurses were predominantly tied to cases that presented with issues beyond healthcare such as legal, financial, or social that necessitated communication and collaboration across multiple care providers.

**Conclusions:**

Care institutions need to adapt to changing and increasingly complex care needs that necessitate cooperation between organizations within and across the health and social sectors. A key facilitator for care coordination and the adequate service provision for complex care needs are multidisciplinary institutional networks, which often remain informal, leaving nurses in the role of petitioner without equal footing. Embedding Case and Care Management in the community has the potential to fill this gap and facilitate flexible, timely, and coordinated care across multiple care providers.

**Supplementary Information:**

The online version contains supplementary material available at 10.1186/s12912-021-00775-0.

## Background

People are increasingly living with complex care needs, characterized by multimorbidity, polypharmacy, issues with mental health and chronic illnesses, and social challenges [1, 2]. In addition, population aging poses a significant challenge to healthcare systems as it relates to increased prevalence of multiple chronic illnesses and questions of long-term care [3, 4]. The healthcare systems in Europe are currently highly fragmented and mostly focused on single diseases [5]. This type of care often does not meet the needs of those affected, as multiple diseases and complex socio-legal and socio-institutional problem situations require patient-centered holistic care [5]. The problems addressed by our study are threefold and elaborated on in the coming paragraphs: a) limited research exists on the interdisciplinary care needs of people living and receiving nursing care in their own homes, b) limited research exists on the increasingly complex realities faced by nurses and the additional skills necessary to work in mobile nursing, and c) to evaluate the need for and potentials of a complex intervention (specifically Case Management).

### Population dynamics and health services

Much of the care and support in Austria as well as much of Europe has traditionally focused on inpatient or hospital care, although reforms are taking place to strengthen the primary and outpatient care sectors [5, 6]. However, social and long-term care is still predominantly provided by family caregivers (42–53%) or mobile home care (32%) [6, 7]. Of those receiving care in their own homes, most suffer from multiple illnesses or have complex care needs that cross the boundaries of healthcare into social, financial and legal care needs [2]. Even though the population‘s needs are increasingly complex, most service providers focus on one illness or on providing a specific service [1], a circumstance that refers in particular to the fragmentation of the system and a lack of multiprofessional teams with the capacity to address socio-legal and socio-institutional problems [5]. Schmidt and Kraehmer [8] propose the implementation of a monitoring system for a network of regional providers as a means to provide needs-based coordination of care and to strengthen the support mechanisms for those with care needs living in their own homes.

Although care at home is increasingly important, underlined by the Austrian strategy towards outpatient care as well as the high amount of informal and formal home caregiving [6], limited research exists on the interdisciplinary care needs of the population [9].

### Care provision and changing work realities

Berntsen et al. [10] and Larsen et al. [11] argue that effective interventions must ensure needs-based adaptibility and flexibility. In addition, continuity of care is considered a key aspect for providing high-quality care in the mobile care setting, and contributes to an overall sense of connected and coherent care over time [12]. The continued management of care across providers, and consistent, uninterrupted direct service provision are considered two main elements of continuity of care [13]. Communication is an important facilitator of continuity and ranges from the relationship between the provider and the client, to the communication and cooperation practices between providers [14]. In order to ensure care continuity for clients, nursing providers must recognize and adopt a number of practices. First, investing in more networked systems of care as a way of providing more holistic care to people living in the community [8]. Second, recognizing that rigid systems as well as rigid players make adaptability difficult, and that there is a need for targeted change management strategies that can facilitate interventions that support complex cases [10, 11]. Third, previous studies have found that nurses facing complex care needs in home healthcare may need to develop of skills and competencies, and expand their psychosocial skills as well as collaborate more closely with social workers in order to provide more holistic care [15, 16]. To date, however, limited research exists on the tasks and complex realities faced by nurses in the home-care setting as well as on the skills necessary for interdisciplinary care [15].

### Complex interventions

Case and Care Management (CCM) offers a two-pronged approach to supporting those with complex care needs living in their own homes [16]. Case Management (CsM) is defined as individualized care tailored towards the client’s needs („package of care“) that is delivered over the entire course of illness or need („over time“) and across institutions, disciplines, sectors and professions („across services“) [17, 18]. Care Management (CrM) takes a systemic approach, builds upon CsM and is defined as system and care planning which coordinates and organises demand-based support across cases and institutions in the social and healthcare system [19, 20]. The focus of CrM lies on providing the population, not the individual affected person, with adequate services and is supplementary as well as additive to the CsM approach [17, 20].

CCM can therefore be regarded as a holistic concept led by the guiding principle that interdisciplinary, professional actions should be designed and implemented in a way that take the individual service needs and respective household and family situation into account [8]. If designed and implemented appropriately, CsM services can provide numerous benefits to service recipients. You et al. [21] conclude that community CsM interventions can significantly improve mental health outcomes as well as reduce unmet service needs. Effects of community based CrM on carer well-being, however, remains inconclusive [21]. A randomized control trial found that nurse-led, multidisciplinary interventions for elderly people residing in their own homes can have benefitial effects on functional performance and well-being [22]. Key components of the intervention were home-visits and individualized, multidisciplinary treatment plans [22]. Additional benefits of CsM have been identified as increased patient satisfaction [23], higher quality of life [24], as well as reductions in hospital stays, rehospitalisation, and the number of days in hospitals among adults with chronic illnesses [25–27]. What is integral to any integrated, community-based care intervention targeted towards meeting complex care needs is needs-based adaptibility and flexibility as well as an interdisiplinary care network [10, 11]. Smith et al. [28] stress the importance of integration among practitioners asll we as on a systemic level Key challenges in the implementation of CsM services are that primary care and community interventions targetting complex conditions are difficult to design and implement [28] and often differ in their design across settings, which contributes to the difficulty of adequately comparing and evaluating their effectiveness [25, 27]. Although the evidence on the efficacy of CsM is inhomogenous, the importance of community based service provision is unquestionable and regarded as a critical cornerstone of primary care.

CrM is currently being rolled out to the entire municipality, with generally positive feedback from institutions and clients alike. However, community care in the form of CsM, as defined above, currently does not exist in Tryol. Rather, mobile nurses are only qualified to provide a series of single services, as opposed to a package of care [29, 30]. In light of changing demographics and increased care and support complexity, the individual services provided by mobile care nurses and health professionals are often inadequate to address the clients‘needs that go above traditional nursing care and include issues of financial, social, mental health, and legal importance best adressed by specialists [31]. Care compelxity is further exacerbated by the expectations of clients and family caregivers towards mobile nursing providers [31]. The lack of CsM also poses a potential threat for the effective implementation of CrM, since CCM is considered a holistic approach that is most successful through the interplay of CsM on the ground and the systemic approach of CrM.

The aim of the study was to evaluate the need for CsM in mobile care organizations by investigating the evolution of mobile care nurses‘task profiles and the challenges in working in a dynamic field with changing target groups and complexifying care needs.

The following four research questions formed the basis of the study and guided the research process:

- Which changes in patient needs are noticed by mobile nurses and which are perceived as especially challenging?
- How is interdisciplinarity and cooperation across care institutions and partners lived and perceived by mobile nurses?
- Which innerinstitutional resources do mobile nurses draw on in the care of people with complex care needs?
- What is the need for case management services as perceived by mobile nursing care organizations and discharge managers?

## Methods

The following study examines the complex care needs of home care recipients in Austria and the expectations levied towards home care nurses. It also investigates the competence needs at the individual nurse and institute levels as they compare to the CCM framework.

### Research design

A qualitative focus group study with a reductive-interpretative data analysis approach was conducted between August and September 2019 [32, 33]. A semi-structured focus group guide was designed to moderate and facilitate the discussions between caregivers. The design was chosen to achieve an in-depth understanding of the individual and institutional restrictions and challenges of caring for people with complex care needs living in their own home. This method is based on the principles of communication, openness, familiarity, unfamiliarity, and reflexivity [34]. Derived from evidence-based literature on integrative CCM strategies, a semi-structured focus group guide was designed in collaboration with registered nurses and public health experts according to the SPSS method (collecting, testing, sorting, and subsuming prompts and questions in a systematic way) [35]. The focus group guide for each professional group can be found in the supplementary materials (Appendix A - C).

### Setting and participants

The included mobile care providers are situated in a municipality defined by its mountainous terrain and remote villages in the Austrian province of Tyrol. Mobile care instutions are an integral part of care provision within the municipality, which struggles with a shortage of specialized doctors, long-term care facilities working at capacity, and a growing aging population. As part of mobile care, nurses travel to their client’s homes to provide the necessary nursing care, with driving times of up to 1.5 h or more, due to remoteness and weather or road conditions.

A purposive sampling approach was applied, meaning the sample criteria and plan were defined based on the theoretical and practical context as well as on the participants’ ability to provide rich and diverse information on the subject matter [34]. The managers of the municipal mobile care institutions were contacted, informed of the study, and asked about their willingness to participate. In order to understand the research context from multiple perspectives, three professional groups (mobile nurses, managers of mobile organizations, and discharge managers) were included in the study. Discharge managers of the local hospital were included in the study to provide insight into interdisciplinary practices in the municipality, and to outline the practice of organizing home care remotely. The criteria for participation were a) a willingness to speak about the challenges of nursing and interdisciplinary work in a home care setting, b) being a registered nurse (RN), discharge manager (DM), mobile home care manager (MHCM) or head nurse (HN), and c) employment at one of the mobile care institutions or the hospital in the municipality. Exclusion criteria were other professions (such as assistant nurses, psychologists, …) and persons working in municipalities without CrM.

### Data collection

All focus groups were conducted at the participants’ workplace and without overlap of institutions or occupational groups, meaning the management level (MHCM and HN) of one organization participated in a separate focus group from the management level of the second organization. Focus groups were always led by one of three researchers (AG, AF, or ES), with a second researcher (AG, AF, or ES) present to provide prompts, take notes, and ensure completeness. The focus groups began with a short welcome and introductory round to ensure a comfortable atmosphere. The used focus group guide included three main sections targeted at understanding the complex realities faced by nurses providing home care, interdisciplinary working practices and networks, and nurse and manager needs towards providing high quality care to people with complex care needs. The complete focus group guide for each occupational group can be found in appendices A-C.

A total of 11.4 h of material was recorded with focus groups lasting between 62 and 76 min, resulting in 299 pages of transcriptions. In order to ensure sample anonymity as much as possible, only data on occupation-specific characteristics (such as position and work experience) was collected.

Data saturation, defined as the point at which additional focus groups no longer yield novel information or repeat what was discovered in previous data collection, and the data provides maximum information on the phenomenon [36], was achieved after six focus groups. For completeness and to ensure every participant had a chance to participate in a focus group, we chose to finish continue data collection for three more focus groups. This was also to ensure that any regional differences within the municipality, such as size of the organization and catchment area, and remoteness and healthcare services, that might have an effect on the the challenges faced by nurses could be taken into account. Data collection and analysis were conducted iteratively in order to explore previously unidentified concepts that emerged during the conversations.

### Data analysis

The recorded focus groups were transcribed according to the guidelines laid out by Kuckartz [37, 38] and analyzed using qualitative content analysis by Mayring [39]. The transcripts were read several times to achieve data familiarization. The software MAXQDA 2020 was used to conduct the data analysis. A reductive-interpretative data analysis approach was taken. The focus was placed exclusively on the content verbally stated or described by the focus group participants, which was then reduced, summarized into categories, and interpreted [40].

Code units were extracted and labelled with a code according to the research aim. The code units were compared and grouped into subcodes according to similarities or differences. The subcodes were reviewed, defined and redefined during the analysis process, according to the research aim as well as reflections by the research team. This approach was meant to ensure the inclusion of the researchers‘ experiences and understanding of the subject matter and the openness towards the content required for qualitative research. The final analysis yielded a total of three main categories, presented with illustrative quotations from the focus groups below. The illustrative quotes are labelled with a code that only indicates the speaker’s profession in order to protect the participants’ integrity.

### Ethics

The study obtained ethical clearance from the Research Committee for Scientific Ethical Questions at UMIT TIROL -Private University and conforms to the standards of informed consent and confidentiality. All research methods were performed in accordance with the relevant guidelines and regulations set out by the Research Committee for Scientific Ethical Questions. Letters of information were sent to the managers and head nurses of the five mobile care services in the municipality and the discharge managers at the local hospital requesting their and their nurses‘participation. All participants were informed about the research, its aims, and their rights as participants in written and oral form before they gave their written consent. All information is handled with strict confidentiality and all identifying information has been removed or anonymized to protect participants.

The entire analysis process was documented, discussed and agreed in the research group at every phase of the analysis process to increase the trustworthiness and thus the credibility and transferability of the results [41, 42]. Credibility was ensured through prolonged engagement with the research context which included multiple meetings with stakeholders in health and social care and the mobile care organizations to build trust and familiarize with the context prior to beginning with recruitment and data collection. In addition, data triangulation, gathering data at different times, from varying locations and organizations, as well as from professionals of differing seniority, and investigator triangulation, where at least two researchers were always involved in the coding, interpretation, and discussion of findings, contributed to credibility. We have aimed to provide a thick description of the setting, participants, and methodology, including the interview guides, within the boundaries of this paper to facilitate transferability judgements [41].

## Findings

A total of twenty-four (*n* = 24) practitioners participated in nine focus groups. Table 1 outlines gender distribution as well as work experience (in years) of the focus group participants.

**Table 1:** Participant Gender & Work Experience

Participant Group	Total (***n***=)	Female (***n***=)	Male (***n***=)	Work Exeriences (Range in years)
Mobile Home Care Managers	5	4	1	3–20
Head Nurses	5	5	0	0.5–9
Discharge Managers	3	2	1	1–5
Registered Nurses	11	11	0	1.5–20

The focus groups resulted in three main categories: the complexity of the case, innerinstitutional frameworks, and interinstitutional collaboration. Each category has multiple subcategories indicated in bold and illustrative quotes that ground the findings in the data are displayed in cursive. The quote by RN2 outlined below exemplifies the nurses’ struggle with providing care to those with complex care needs. It outlines not only the resource intensity of integrating external partners into the care process, but also hints at tasks that go beyond the nurse’s capabilities and current skill level.

*That is what makes it so complex, because you need multiple contact people. We don’t have a problem with providing nursing care, we do that ourselves. But with everything that doesn’t pertain to nursing, I need someone else and that is what makes it complex and time intensive. (RN 2).*

### The complexity of the case

Participants indicated that the complexity of a case is dependent on a large range of characteristics associated with the client, whereby the number, interplay, and area of the characteristics contribute to the level of perceived complexity. The complexity characteristics were split into five subcategories that include the physical health state of the client, psychological and mental health of the client, the social embededdness of the client, the financial and environmental circumstances of the client, and uncertainty and worries that pertain to the client.

#### Physical state of health

Challenges in the physical health state of the client arise in the large variety of different illnesses the carers are confronted with, multimorbidities, general physical decline due to disability or age, and the provision of palliative care.

*We nurses usually work completely alone and autonomously with elderly people or those in a palliative state. But that isn’t enough. We urgently need the professional support of multiple healthcare institutions coordinated by a case manager. (RN 6).*

#### Psychological and mental state of health

Nurses indicated feeling overwhelmed when confronted with certain psychological and mental health issues, especially in the context of the client’s own home. The most commonly referenced reason for discontinuing a client’s care from the side of the service provider was an underlieing psychological or mental health issue. Aggression was stated among one of the most challenging problems faced by nurses in the provision of at home care with RN 2 stating that nurses are faced with “*cases of aggression or behaviors that are neither safe nor acceptable for us to work with*”. Addiction, related behavioral patterns, and corresponding organic brain damage where “*it is not about curing the illness but rather stabilising the affected person*” (MHCM 4) are named as additional challenges. Hoarding, self-neglect and compulsive disorders are identified as particularly disruptive because they carry significant financial and resource consequences for the carers as well as the affected individual.

*For a long time I have experienced a very stressful situation with a client, whom I have been continuously supporting for three or 4 years. This client has already been evicted multiple times. We have already done a lot for him, supported him financially, helped with cleaning and decluttering, the client is a compulsive hoarder – even helping to clear out, vacate and clean the apartments. (RN 11).*

#### Social embeddedness

In the context of the client’s social embeddedness, nurses indicated that clients without relatives or informal caregivers were difficult to care for because the lack of a social network often contributed to the client‘s loneliness and all care activities had to be assumed by the nurses. This was especially difficult for nurses, when they could not guarantee their client’s safety after they left due to other client characteristics such as detereorating physical state or underlieing mental health issues. The informants also indicated feeling overwhelmed when the clients have family or relatives who show little to no interest in the client’s wellbeing or care. In addition, according to the participants a lack of social network and informal caregivers contributed to self-neglect, poor hygiene, and general squalor.

*Unfortunately, there are clients that either do not have relatives or, even worse, that have relatives who show absolutely no interest iin them. That is really awful. (RN 4).*

#### Financial and environmental circumstances

The client‘s financial and environmental circumstances also contribute to the complexity faced by home care nurses, especially since they often lack the further education necessary to adequately handle the case. Evictions or impending evictions pose a challenge, due to the large number of stakeholders and the organization necessary to address the issue. Homes without modern sanitary facilities, a circumstance still found in some few rural Austrian homes, were named as a challenge as well. Identifying, organizing and enabling access to financing possibilities seem to be a challenge for home care services.

*Yes, there are often situations where we do not have a solution ready. For example, when someone does not have a residence or loses their residence during their hospital stay. Then it becomes extremely difficult for us, because we cannot search for an apartment or are overwhelmed by the situation.*

*(DM 1)*

#### Uncertainty and worry

Lastly, the informants mentioned their struggle with leaving clients alone in their own homes after providing care and support and having to accept the uncertainty associated with that. Institutional, financial, human resource, and time constraints contribute to the inability to ensure the client’s safety outside of the care appointment setting.

*I remember a 50 year old client living alone with highly complex nursing needs. My colleagues and I had a bad feeling every time we left her alone after providing care. She was alone for most of the day. (RN 5).*

### Innerinstitutional frameworks

The identified innerinstitutional framework was classified into two subcategories: the expansion of care tasks beyond nursing care and the working hour model used in mobile home care organizations.

#### Care tasks extending nursing care

The informants found that the services expected to be delivered by mobile home care organizations, which were traditionally exclusively nursing tasks, increasingly go beyond nursing and include aspects of the healthcare and social sectors. Participants stated that targeted further education in social, financial and legal matters would be vital, since basic nurse training content does not cover the necessary depth nor convey practical guidance.

*“To be completely honest, we as nursing care providers are not equipped with the tools to provide adequate social work and consultations.” (HN 2).*

#### Working hour model

Mobile home care organizations have a high proportion of part-time employees. Informants stated that, on the one hand, this enables flexibility for the nursing staff and management, contributes to general job satisfaction and is considered family-friendly. On the other hand, this complicates the continuity of care across multiple service providers, especially as soon as calls to external stakeholders are necessary, follow-ups are initiated or networks must be maintained. MHCM 1 describes it as a “*big problem concerning information transfer, where as a result, a lot of information regarding the clients gets lost*”. The HNs and MHCMs added that investments in further education for part-time staff is difficult due to the high turnover rate and limited time spent at the workplace.

*“We need more specialised training, but freeing registered nurses up and financing further education is difficult without subsidisation by the regional government” (MHCM 2).*

### Interinstitutional collaboration

According to the participants, constructive cooperation between the individual network partners in the health and social care systems (in particular the general practitioners, senior and nursing homes, and discharge managers of the hospitals) is of major importance for the provision of high-quality, individualized care to people with complex care needs. There were, however, a number of differences in collaboration practices between the different stakeholders which is why subcategories were formed base on stakeholder groups.

#### General practitioners

The cooperation between the mobile home care organizations and general practitioners was characteritzed by active disclosure of information, a passive exchange of information, and mutual respect of the working relationship. General practitioners play a very important role in the care of people living at home in the region. Both positive and negative collaborative efforts between the mobile home care organizations and general practitioners were identified. The passing on of information as well as the willingness to share information was stated to be essential. The informants found it particularly positive to work with those general practitioners who, after a home visit, actively impart relevant information about treatment changes to the mobile home care organization in charge of care. Especially in view of the part-time working hour models, obtaining information from general practitioners if often difficult and calls remain unreturned unless they are centralized at the mobile home care organization.

*„ … I find that some of the general practitioners in our region are difficult sometimes. I always have to go get something, I have to ask, I have to call, or when they change the treatment or a medication, it is really difficult for me when they don’t say anything or tell me.” (RN 4).*

The informants expressed the strong wish to work with the general practitioners at eye level. The informants stated that they feel that the potential for relieving general practitioners‘workload and burden, and the care provided by the mobile home care organizations is often undervalued by the general practitioners. No clear strategy for strengthening the network and collaboration between the mobile home care organizations and general practitioners exists and is often based on existing cooperations or informal exchanges.

*“With the doctor, we often have the feeling, he is the doctor, he knows best what the patient needs, and he judges us. … I think, it must be his ego.” (HN 3).*

#### Senior and nursing homes

Collaboration with the senior and nursing homes is characterized by joined decision making and close collaboration to ensure that decisions on placements are made on a needs-basis. According to the interviewed employees of the mobile nursing and care organizations, the cooperation between mobile nurses and nursing homes has become increasingly more positive in recent years. According to the informants of the mobile home care organizations, they are often asked for a needs assessment of their clients when a nursing home spot becomes vacant which contributes to the feeling that mobile nurses are considered equal partners in the care provision process.

*Then they call us and ask us about our opinion regarding the patient’s situation. That is great. They drive to the patient’s home and see which patient has the highest need for the space in the nursing home. I really feel that the patient with the most urgent need is also the one to receive preference nursing home placement. (RN 2).*

From the participants’ point of view, the search for a free place in a retirement and nursing home is often time-consuming and does not always promise success. While organization is part of a head nurse’s job description, the shortage of nursing home spots leaves the nurse as a petitioner without equal footing.

*I start by calling the nursing homes closest to the client. If there are no vacancies, then I ask at the second geographically closest nursing home. If I still haven’t found a vacancy, then I proceed according to kilometer-distance to the client’s home. (HN 3).*

## Discussion

People are increasingly living with complex care needs, characterized by multimorbidity, polypharmacy, issues with mental health and chronic illnesses, and social challenges [1, 2]. In addition, population aging poses a significant challenge to healthcare systems as it relates to increased prevalence of multiple chronic illnesses and questions of long-term care [3, 4]. The aim of the study was to investigate the need for CsM in mobile care organizations by investigating the evolution of mobile care nurses‘ task profiles and the challenges in working in a dynamic field with changing target groups and complexifying care needs. We found that case complexity is driven by patients’ physical and mental health needs, financial and social environment, as well as difficulties dealing with uncertainty. Innerinstitutional issues lead to loss of information such as the lack of expertise in areas beyond nursing as well as the high percentage of part-time nurses. This could be adressed through additional training as well as a person with a coordinative function. Interinstitutional collaboration is characterized by ambivalence, with informal network structures based on personal attitudes rather than cooperation on equal ground that is largely dependent on the stakeholder in question.

### Case complexity

This paper provides an ammendment to Andersson et al.’s [15] call for additional research into the complexities faced by nurses working in a community setting and the task profiles expected of them. At the case level, the client’s physical and mental health status as well as their social embeddedness and financial means contribute to complexity.

One of our main findings confirms Andersson et al.’s [15] statement that providers are focused on supplying single forms of care. Berntsen et al. [10] and Larsen et al. [11] argue that interventions must ensure needs-based adaptibility and flexibility. In our study, providers are aware of changing population needs and how those affect service provision, however are unable or unwilling to change their structures, often due to the structural and systemic framework in which they work. In order to change mindsets within organisations as well as provide appropriate training in complex care, a structured curriculum in CCM can provide the plattform for a paradigm shift within existing structures. Smith et al. [28] argue that interventions should not solely focus on integration among practitioners but also on integration into the systemic framework within which they act. In our case, structural changes at the systemic level are carried out through targeted actions by care managers in order to further facilitate the change process. As Duncan [43] points out, joint care planning and support as well as collaborative organisational development recognizes the overlapping nature of mental, physical and social well-being and recognizes the vital contribution of community nurses to the functioning of community care and population health outcomes.

At the innerinstitutional level, researchers discovered an awareness among RN, HN and MHCM of the changing and increasingly complex service environment, yet also an underlying reluctance towards adapting their provided services to these changes. Researchers identified the need for further education among nurses, especially in the areas of mental health and social care. Further education for nurses should provide training in the areas of psychosocial and complex care as well as the provision of integrated, networked care, especially with other disciplines such as social workers [44].

Interinstitutional frameworks in the form of more networked systems are an approach to provide better bio-psycho-social care to people living in the community [8]. A network of care providers with rigid systems as well as rigid players, however, make adaptability difficult and therefore there is a need for targeted change management strategies that can facilitate this transition [10, 11].

Continuity of care is considered a key aspect for providing high-quality care in the mobile care setting: managing care across providers and consistent, uninterrupted direct service provision are considered two main elements [13]. A multidisciplinary review by Haggerty et al. [12] postulates that there are three types of continuity, informational, relational, and management, which contribute to an overall sense of connected and coherent care over time. Communication is an important facilitator of continuity and ranges from the relationship between the provider and the client, to the communication and cooperation practices between providers [14]. In order to provide continuity of care for people with complex care needs, the care setting, the institutional framework as well as collaboration practices between institutions are integral.

Researchers found that a lack of formal network structures, a reluctance to adapt services and provision practices, as well as an absence of continuous evaluation using defined indicators within and across institutions may contribute to difficulties in providing continuous care to clients with complex care needs. Recommendations for nursing practice are the development of patient and care outcomes for care within and across service providers, a formalized network structure with pre-defined responsibilities across caregivers, as well as continued education and training for nurses targeted at effectively caring for people with complex care needs [45]. Combining nursing education and training with formal network partners will alleviate strain on nurses as they will have the ability to recognize when and which support to recruit when necessary and ensures continuity of care for patients. The function of a CsM is to facilitate interorganisational collaboration and may play a crucial role in improving continuity of care in mobile nursing organisations [46].

According to the Kaiser Pyramid Model, patients with comlex care needs account for only 5% of the population, yet consume up to 60% of health resources [47]. Care structures close to communities and the lived surroundings of people with complex care needs provide vital and low-threshold support. Results of this study, however, underline the disconnect between the implemented CrM at the systemic, regional level and the mobile care institutions on the ground, due to a lack of a dedicated CsM that functions as a formal point of contact within and across institutions and for CrM, and is equipped with the skills and know-how to support complex cases. Studies in an international context, such as by Duarte-Climents et al. [48] on Community Liaison Nurses in Spain that apply a CsM approach, found that CsM techniques can lead to improvements in patients’ clinical conditions as well as in the quality and efficiency of care.

Countries, such as Austria, that are considered to have weak primary care structures [49] should consider establishing service positions close to the community using CsM techniques in order to improve access to, continuity of, and quality of care, and to support mobile care organizations working on the ground. However, a challenge remains as mobile care institutions themselves recognize changing needs, but are reluctant to adapt their servie patterns and have no dedicated case management as contact persons for the formatlized network. Through a CsM approach, a contribution can be made to health equity by making health-promoting services and structures accessible to all at low thresholds and close to home.

### Strengths & Limitations

In an effort to limit bias, researchers remained conscious of the study’s aim and research questions when conducting the interviews and data analysis. Since interviews were conducted by three different researchers, a certain amount of interviewer bias can be expected. However, much of this bias is addresed by the presence of two interviewers at all times. The main strength of the study lies in the large number of nurses, as well as all head nurses and mobile healthcare managers from the institutions within the municipality that agreed to take part in the study, allowing for a wholesome viewpoint of the subject matter. However, only one municipality was included in the study due to its unique characteristics concerning the implementation stage of CrM. Qualitative content analysis was used to identify key categories of text with only some differences between the organisational levels explored. Focus groups included an uncharacteristically low number of participants, which was due to the small size of most mobile care organizations in the municipality as well as the fact that mobile nurses shift scheduling did not allow us to meet with the entire staff at the same time. However, we were still able to facilitate and moderate a rich discussion. This study’s findings cannot be generalized; however, parallels can be drawn to similar contexts.

## Conclusion

In order to provide continuous, high-quality care for people with complex care needs living in their own homes, mobile care organizations need to adapt to changing and increasingly complex care needs that are more resource and time consuming than traditional nursing tasks. These complex care needs necessitate cooperation between organizations within and across the health and social sectors, whereby nurses are in an ideal position to identify client needs and drive care coordination. The reluctance of the mobile care community to do so is an attest to a fragmented system and a sense of helplessness in a system that has long become too complex, fragmented, and polarized to navigate with any ease. Nevertheless, nurses and managers are comitted to providing care at the highest possible level and often go above and beyond in order to do so. We recommend nursing organizations take the initiative to recruit and formalize cooperation agreements with external partners in health and social care in order to ensure collaboration on equal footing.

Further research into the network perspectives of other service providers such as general practitioners and pharmacies is necessary, in order to understand the barriers to implementing an integrated network structure for collaborative and continuous care. In addition, research that incorporates the viewpoint of those receiving care is needed.

## Supplementary Information

**Additional file 1 Appendix A** Interview Guide for Registered Nurses. This file contains the interview guide used for the focus groups conducted among registered nurses. The original guide was in German and translated into English for dissemination. **Appendix B** Interview Guide for Mobile Home Care Managers & Head Nurses. This file contains the interview guide used for the focus groups conducted among mobile home care managers and head nurses. The original guide was in German and translated into English for dissemination. **Appendix C** Interview Guide for Discharge Managers. This file contains the interview guide used for the focus groups conducted among discharge managers at the local hospital. The original guide was in German and translated into English for dissemination.

## Data Availability

The raw data is not shared to protect the participants’ integrity, however it can be made available from the corresponding author upon reasonable request.

## Abbreviations

CCM: Case and care management
CrM: Care management
CsM: Case management
DM: Discharge manager
HN: Head nurse
MHCM: Mobile home care manager
RN: Registered nurse
