# Supplementary material for: Demand for community-based Case Management in Austria - a qualitative analysis
Source: BMC Nurs. 2022 Jan 4;21:5. doi: 10.1186/s12912-021-00775-0 (PMC8725559; doi:10.1186/s12912-021-00775-0)
Supplement: Supplementary file 1 — Additional file 1 Appendix A Interview Guide for Registered Nurses. This file contains the interview guide used for the focus groups conducted among registered nurses. The original guide was in German and translated into English for dissemination. Appendix B Interview Guide for Mobile Home Care Managers & Head Nurses. This file contains the interview guide used for the focus groups conducted among mobile home care managers and head nurses. The original guide was in German and translated into English for dissemination. Appendix C Interview Guide for Discharge Managers. This file contains the interview guide used for the focus groups conducted among discharge managers at the local hospital. The original guide was in German and translated into English for dissemination. [file 12912_2021_775_MOESM1_ESM.docx]

**Demand for community-based Case Management in Austria - A qualitative analysis**

**Supplementary Materials**

Alessandra Schirin Gessl BSc., MA., Angela Flörl BScN**, Assoc.-Prof. Dr. MMag. Eva Schulc

Division for Integrated Care, Institute of Nursing Science,

Department of Nursing Science & Gerontology

Eduard-Wallnoefer Zentrum 1, 6060 Hall in Tyrol, Austria

UMIT TIROL - Private University for Health Sciences, Medical Informatics and Technology

**Corresponding author: Flörl, A. (angela.floerl@umit.at)

## Appendix A – Interview Guide for Registered Nurses

1. **Demand for further development of case management in mobile community care organizations**

1: Please tell us which tasks you perform within the framework of the existing case management (individual services of the service catalog)?

2: Which cases (target group, context), for which you perform case management, do you experience as stressful?

3: What suggestions for improvement do you have to optimize the existing case management?

4: Where do you see additional need for further development and/or optimization of the existing Case Management?

1. **Interfaces (hospital, general practitioners, long-term inpatient facilities) and processes**

5: How do patients access your organization?

6: How are clients admitted for care at the mobile community care organization?

7: What did you do if in the past you needed support when caring for patients with complex care needs (internal, hospital, family doctors, inpatient long-term care facilities)?

8: How do you see the cooperation with the professional groups involved (hospital, general practitioners, inpatient long-term care facilities)?

9: Would additional network meetings between the professional groups involved be helpful?

10: How do you contribute to ensure sustainable cooperation between professional groups (network care)?

11: When thinking of other professional groups, is there anything else you would like to say about the cooperations?

12: How does the transfer or discharge of a client take place in your organization?

1. **Quality requirements within the existing case management**

13: What measures do you take for quality assurance?

14: Which actors from the professional groups involved are actively involved in the evaluation process?

15: Have you received feedback (patients, nursing and care organizations, etc.) regarding the case management that has taken place?

1. **General Description of Case Management**

16: Would you describe the tasks of case management, as they are defined today,

1. as part of the nursing process and/or

2. as ist own process and/or

3. as a collection of single services?

Please explain your answer.

1. **Further Education**

17: What further education have you attended?

18: Are you interested in further education in the field of Case and Care Management?

1. **General Information about the Interviewee**

19: When did you complete your education as a registered nurse?

20: How long have you been working as a nursing care professional in the mobile setting?

1. **Final Remarks**

21: Is there anything else that is important to you but has not yet been addressed?

22: Do you have any questions for us?

## Appendix B – Interview Guide for Mobile Home Care Managers & Head Nurses

1. **Demand for further development of case management in mobile community care organizations**

1: Please tell us which tasks you perform within the framework of the existing case management (individual services of the service catalog)?

2: Which cases (target group, context) for which case management is performed does your staff experience as stressful?

3: What suggestions for improvement do you have to optimize the existing case management?

4: Where do you see additional need for further development and/or optimization of the existing Case Management?

1. **Interfaces (hospital, general practitioners, long-term inpatient facilities) and processes**

5: How do patients access your organization?

6: From your point of view, how are clients admitted for care into the mobile community care organization?

7: What did you do if in the past you needed support when nursing and caring for patients with complex care needs (internal, hospital, family doctors, inpatient long-term care facilities)?

8: How do you see the cooperation with the professional groups involved (hospital, general practitioners, inpatient long-term care facilities)?

9: Would additional network meetings between the professional groups involved be helpful?

10: How do you contribute to ensure sustainable cooperation between professional groups (network care)?

11: When thinking of other professional groups, is there anything else you would like to say about the cooperations?

12: How does the transfer or discharge of a client take place in your organization?

1. **Quality requirements within the existing case management**

13: What measures do you take for quality assurance?

14: Which actors from the professional groups involved are actively involved in the evaluation process?

15: Have you received feedback (patients, nursing and care organizations, etc.) regarding the case management that has taken place?

1. **General Description of Case Management**

16: Would you describe the tasks of case management, as they are defined today,

1. as part of the nursing process and/or

2. as ist own process and/or

3. as a collection of single services?

Please explain your answer.

1. **Further Education**

17: What further education have you attended (if you administer case management)?

18: Are you interested in further education in the field of Case and Care Management (if you administer case management)?

1. **General Information about the Interviewee**

19: What is your educational background and when did you complete your education?

20: How long have you been working as head nurse or mobile community care manager?

1. **Final Remarks**

21: Is there anything that you feel is important but has not been addressed?

22: Do you have any questions for us?

## Appendix C – Interview Guide for Discharge Managers

1. **Status Quo of the Discharge Management**

1: Please tell us which tasks you perform as a discharge manager.

2: Which assessment tools do you use as part of your work as a discharge manager?

1. **Processes and Interfaces with mobile community care organizations**

3: What is the discharge process for patients into mobile community care organizations?

4: In which cases (context, target group) is discharge into mobile community care difficult?

5: What did you do if in the past you needed support when caring for patients with complex care needs (internal, mobile care organization)?

6: How do you see the cooperation with the mobile community care organizations?

7: What suggestions do you have for optimizing the existing cooperation with mobile care organizations?

8: Would additional network meetings with the mobile community care organizations be helpful?

9: How do you contribute to ensure sustainable cooperation with the mobile community care organizations (network care)?

1. **Quality requirements within the existing discharge management**

10: What measures do you take for quality assurance?

11: Are the mobile care organizations actively involved in the evaluation process?

12: What actions can you take if a patient discharge is scheduled for a Friday?

13: What are your expectations for a case manager in the community?

14: Do you receive feedback from patients, care organizations, etc. about the discharge management process?

1. **Aus-, Fort- und Weiterbildungen**

15: What is your educational background (e.g. registered nurse) and when did you complete your education?

16: What further education have you attended?

1. **General Information about the Interviewee**

17: How long have you been working as a discharge manager?

18: What is your approximate shift schedule?

19: How many patients do you work with on an average day?

1. **Final Remarks**

20: Is there anything that you feel is important but has not been addressed?

21: Do you have any questions for us?
